# Overcoming Obstacles to Develop High-Performance Teams Involving Physician in Health Care Organizations

**DOI:** 10.3390/healthcare9091136

**Published:** 2021-08-31

**Authors:** Simon W. Rabkin, Mark Frein

**Affiliations:** Division of Cardiology, University of British Columbia, 9th Floor 2775 Laurel St., Vancouver, BC V5Z 1M9, Canada; markfrein70@gmail.com

**Keywords:** high performance teams, physician engagement, physician burnout, health care teams, health care leadership, health care culture

## Abstract

Many health care organizations struggle and often do not succeed to be high-performance organizations that are not only efficient and effective but also enjoyable places to work. This review focuses on the physician and organizational roles in limiting achievement of a high-performance team in health care organizations. Ten dimensions were constructed and a number of competencies and metrics were highlighted to overcome the failures to: (i) Ensure that the goals, purpose, mission and vision are clearly defined; (ii) establish a supportive organizational structure that encourages high performance of teams; (iii) ensure outstanding physician leadership, performance, goal attainment; and (iv) recognize that medical team leaders are vulnerable to the abuses of personal power or may create a culture of intimidation/fear and a toxic work culture; (v) select a good team and team members—team members who like to work in teams or are willing and able to learn how to work in a team and ensure a well-balanced team composition; (vi) establish optimal team composition, individual roles and dynamics, and clear roles for members of the team; (vii) establish psychological safe environment for team members; (viii) address and resolve interpersonal conflicts in teams; (xi) ensure good health and well-being of the medical staff; (x) ensure physician engagement with the organization. Addressing each of these dimensions with the specific solutions outlined should overcome the constraints to achieving high-performance teams for physicians in health care organizations.

## 1. Introduction

There is considerable ongoing interest in the creation of high-performance teams, involving physicians in the health care workplace and in health care organizations [1,2,3,4,5,6,7,8]. Heath care organizations are different from other types of organizations and these differences need to be taken into account in the evaluation of any framework for performance [9]. Health care organizations consist of a number of complex professional bureaucracies that deal with an even more complex set of political, legal, financial, customer (patient), and community challenges [9]. In contrast to most organizations, there is limited managerial control of physicians who, in part generate the workload and costs yet who have loyalty to professional values rather than organizational views [9]. Similarly nurses have loyalty to a professional agenda that influences hospital activities and this adherence can be of more importance to them than a health care organization’s philosophy [9]. These and other differences between health care and other organizations justify the need to examine high-performance teams focusing only on health care organizations and the physician’s role in them.

A team can be defined as a group of individuals who work together for a common goal. A high-performance team consists of individuals with a common purpose, often with different skill sets or perspectives yet with high levels of collaboration and innovation, that produce superior results [10]. While each member contributes to the team, the entire group is responsible for the team’s success. “High-performance teams are characterized by an atmosphere of buoyancy… By showing appreciation and encouragement to others members in the team, high-performance teams create emotional spaces that are expansive and open possibilities for action and creativity… In addition, they accomplish their tasks with ease and grace. In stark contrast, low performance team struggled with their tasks, operated in very restrictive emotional spaces created by a lack of mutual support and enthusiasm, often in an atmosphere charged with distrust and cynicism” [11].

In health care, most teams have been created because of the complexity of the medical problem or condition which requires the input of different perspectives—an assembly of a group with a different knowledge base or skill sets [1]. The reasons for the interest in the incorporation of physicians into health care management to create high-performance teams are many and include that physicians should be able to assist in the allocation of hospital resources to minimize their overuse, underuse, and misuse; in addition, there is a direct relationship between a high-performance work place and the quality of patient care [5,12,13]. Unfortunately, many health-care organizations struggle and often do not succeed as high-performance organizations that are not only efficient and effective but also are enjoyable places to work. It is important to dissect why some health care organizations do not achieve the high levels of innovation and high-performances that have been achieved by some non-health-care corporations. Physicians are an essential part of the health care organization, yet their role in ensuring high-performance teams has been mainly focused on patient care teams and there is much less assessment of their potential role in organization performance.

The objective of this review is to exam health care organizations from the physician’s perspective, in order to synthesize data on why some health care organizations do not meet the metric of a high-performance work place and what is needed to create high-performance teams inclusive of physicians. Ten dimensions were constructed that are obstacles or impediments to achieving high-performance teams for physicians in health care organizations. A thorough literature review was conducted (the search methodology is outlined in the supplement) in order to examine their contribution to teams. The characteristics of high-performance teams have been identified by a number of authorities [14,15,16]. The objective of this review is to examine and validate, if justified, the obstacles for the creation of high-performance teams with physicians in health care and the methods to overcome these obstacles in order to create a high-performance health care organization.

## 2. Obstacles to Develop High-Performance Teams Involving Physicians

### 2.1. Failure to Ensure That the Goals, Purpose, Mission, and Vision Are Clearly Defined

While it is generally recognized that a successful team must have clear goals, the definition or construction of goals is more complex than it appears. In the health care sphere, goals have been classified as immediate ones—synonymous with measurable work output, intermediate goals with measurable project outcomes, and long-term goals that are aimed at a broader impact on the organization [17]. The type of goals vary from performance metrics for workers, to the organization’s economic targets, and to the cultural (social) needs of the organization [17]. Just as the lack of a goal will inevitably lead to a lack of productivity, poorly defined goals will confuse the direction of the efforts. Regardless of the type of goal, it is imperative that the goals are clearly defined, as success is measured by how the identified goals are achieved.

A health care team’s vision or purpose often reflects their institution’s vision or mission statements. Unfortunately, these statements are often abstract, using phraseology about caring for patients and health care providers. Mission statements have been seen as “an expensive expression of politically correct platitudes which leads to cynical alienation of stakeholders.” [18]. Creating an effective mission statement is difficult and for some health care managers it is an extremely frustrating task [19]. Enlisting the physician stakeholders in the creation of the vision/mission statement may be helpful in establishing goals that are relevant to the physician component of the team. Failure to integrate data from all relevant stakeholders has been suggested to omit the significant interests of one or more groups and reduces the effectiveness of any strategic planning process [20]. A review of hospitals (Public, Private and Social Sectors) that were part of the Northern Regional Health Authority of Portugal, found quality and excellence were referred in the mission of all (100%) hospitals but the closest metric to performance, identified was technical efficiency in only 55% of hospitals [21]. Thus, assessment of performance does not appear to be a highly valued component of hospitals’ vision or mission.

Failure to have a clear statement of goals, that are refreshed when needed, will lead to stagnation and will impact the development of high-performance teams.

### 2.2. Failure to Establish a Supportive Organizational Structure That Encourages High-Performance Teams

While it may appear self-evident that an organization’s structure needs to encourage high-performance teams for high-performance teams to succeed [22], organizations are complex and multidimensional and various aspects of the organization at the different levels should be supportive for teams to perform at a high level. If the organization does not value physicians in a complementary role with operations managers and other health professional then a key element of the health care organization will be missing in order to create a high-performance team.

**Figure 1:**
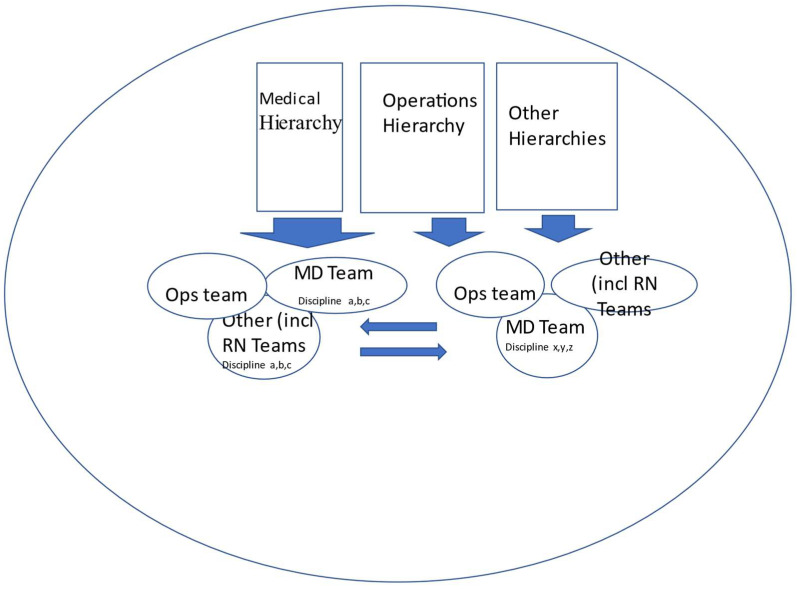
The interactions of a single team within a health care organization.

A team, including physicians may work well together but not achieve high-performance metrics if the other teams with which it interacts are not supportive or if the medical, operations, and other hierarchies within the organization are not supportive of the teams (Figure 1).

Physicians in the health care teams usually belong to a hierarchical professional structure (medicine, surgery etc.,). Health care organizations are complex entities. Senior leadership needs to define the relationship between the team and the relationship between the teams and their “Departments” or the organizational units in which they operate in order to minimize the conflicts or obstacles for attainment of maximum performance. “Tension between corporate management priorities and physicians can revolve around clinical freedom and autonomy” [22]. The organization should not limit an excellent team’s flexibility but rather facilitate it to adapt and improve performance.

In hierarchical health care organizations, the institution’s board of directors bears the ultimate responsibility for institutions’ functioning. In some jurisdictions, boards are not fully meeting their governance requirements for high-quality, safe-care delivery [23]. The data strongly support that physician engagement with their health care institution improves patient safety [24]. The board must act to ensure high-performance teams and ensure physician involvement. These actions focus on an organizational strategy for high-quality care, with the chief executive officer held accountable for evidence of successful implementation and monitored by the board [23].

### 2.3. Failure to Ensure Outstanding Physician Leadership

Outstanding physician leaders are essential to create a high-performance team in health care. Within health care, there are two different kinds of teams—clinical teams involved with direct patient care and management teams involved in establishing guidelines for patient management, resource allocation etc. There are perceived differences in the competencies required for these two kinds of teams [25]. For a clinical team, clinical skills and knowledge are obviously important but in addition the commitment to working collaboratively, commitment to a quality outcome and commitment to the organization are considered essential [25]. In addition, clinical leaders should possess emotional intelligence, resilience, self-awareness and understanding of other clinical disciplines [26]. For management teams, members need to display a strong focus on the organization and its values and culture [25]. Physicians are trained in clinical assessment, clinical decision-making, interpretation of images etc., but much less commonly receive training in leadership. Physicians may fail in a leadership role because of lack of training or lack of mentorship.

While recognizing support for shared/collaborative/collective leadership, high-performance teams require an excellent leader to maintain the focus on goals and priorities [14] and to ensure that individual team members maintain the correct focus [14,27]. Successfully implementing a strategic change often requires getting individuals to change their behaviors. Leaders can enhance the likelihood of successful strategic change within an organization by developing good teamwork [28].

Suitably trained physician CEOs are considered more likely to create a high-performance organization. Goodall correlated physician leadership with an index of health care performance [29]. The index assigned a percentage to a number of different spheres specifically the resourcing of patient care; mortality rates 30 days after admission; the delivery of care; and a “patient-safety index” [29]. The mean score of US hospitals that focused on cancer, digestive disorders, and heart disease, was significantly higher when the chief executive officer was a physician compared to similar hospitals with a CEO who was a professional manager [29]. In an analysis of large US hospital systems in 2015, those led by physicians received higher (*USNWR*) ratings and bed usage rates than did hospitals led by non-physicians, with no differences in financial performance [30]. There have been a number of explanations proposed for these observations and range from the capacity of an “expert leaders” to understand the complexity of their organization to the compassion of a physician to advance a patient and health care provider orientation and increase job satisfaction of all employees [31]. A health care organization can fail to become a high-performance enterprise when/if its physician leadership is not trained in leadership and/or is not frequently assessed for leadership performance.

Other than the CEO position, there are many other areas of hospital management where physicians are in leadership positions such as heads of departments or divisions. Those medical leaders have the challenge of fulfilling both organizational and medical staff objectives [32]. The failure of physician leadership to make a meaningful difference in performance compared to non-physician managers has been noted in some situations [33]. This finding has been attributed to the lack of leadership or teamwork training in medical school education and/or the nature of medical practice that shapes physicians’ attitudes [34]. This kind of failure highlights the need for training of physicians in leadership.

### 2.4. Failure to Recognize That Team Leaders Are Vulnerable to the Abuses of Personal Power or May Create a Culture of Intimidation/Fear with the Development of a Toxic Work Culture

The choice of a team leader or leaders are crucially important for team performance as studies have demonstrated that managerial competencies are positively associated with organizational performance [35]. Physicians placed into leadership positions often lack the necessary skill sets to be a leader of a heath care team. The lack of leadership or teamwork training in medical school education is one factor [34]. Another is the nature of medical practice which is usually a one-to-one relationship with a patient in which the physician is mainly “in charge” of the relationship [34]. Third, a medical practice usually requires physicians to give orders for management decisions rather than work in a team. Fourth, physicians are judged on their individual performance in patient care, image interpretation, and technical skills. Physicians may not readily adapt to team work. It is paramount that physician leaders acquire or develop the unique blend of knowledge and skills that support or lead a team to develop innovative solutions for improvement of their health care organization [27]. Skilled team leadership is essential.

Health care environment can readily become a culture of intimidation which has been identified in countries across the globe [36,37,38,39,40,41,42]. Physicians become afraid to speak out for fear of losing access to institutional resources [43]. Bullying in the medical workplace, is not restricted to only one specialty or level of training; the victim can be a trainee, a physician in practice, or a consultant [43,44]. Bullying can lead to physician mental health problems, reduced job satisfaction, and can compromise patient care [43,44]. The bully is most often a superior, who does not have the capacity to create a safe environment which encourages creative ideas and their expression [39,45,46]. A team with this kind of leader will not coalesce into a high-performance team.

A toxic organizational culture has been recognized to be a major contributing factor of serious failings in health care delivery [47]. Qualitative analyses distinguished two different types (a) (so called) intangible themes (commitment, trust, psychological safety, power, support, communication openness, blame and shame, morals and valuing ethics, and cohesion) and (b) (so called) tangible themes (leadership, communication system, teamwork, training and development, organizational structures and processes, employee and job attributes, and patient orientation) that can identify a health care organization with a toxic culture [47]. Failure to recognize a toxic culture in the entire organization or in smaller components of the organization leads to the destruction of the possibility to produce high-performance teams.

### 2.5. Failure to Select a Good Team and Team Members—Team Members Who Like to Work in Teams or Are Willing and Able to Learn How to Work in a Team and Ensure a Well-Balanced Team Composition

A high-performance team requires team members who like to work in teams or are willing and able to learn to work in a team. Some organizations only accept individuals into a team if the person has proven expertise in working in a team and/or have a good understanding of the organization’s mission, structure, economics, politics [27]. A team member who does not have the interest of the team as a priority or who does not respect others in the team will produce rancor and will inhibit the team from attaining high performance. Just as some physicians will not be good team leaders, some physicians will not be good team members.

In the clinical setting, increased diversity of the team can improve the ability of the team to innovate, communicate to patients, and assess patient risk [48]. Diversity of the team means diversity of age, gender, ethnicity, educational background, etc. Patients generally fare better when care is provided by more diverse teams [48,49,50]. Whether this translates into physician groups in non-clinical activities is presumed but apparently not yet proved.

There have been calls for increased diversity in health care leadership with the expectation that it will translate into higher quality health care [51]. Although evidence to support this claim are sparse in health care, data from industry appear to support this approach [52]. There are a number of learnings/experiences, that have developed successful practices for health care organizations [53]. Diversity in a team, however, can lead to more conflict and lower productivity [50,54]. The other caution about diversity in teams is that even when diversity is good for group performance, it may lead to deterioration in interpersonal relations and attitudes toward the group [49].

Recognizing that there is a need for a more complex conceptualizations and definition of diversity [50], there is a need for maximizing the incorporation of diversity to improve effectiveness while minimizing the attributes that will lead to more conflict and less productivity within medical teams. Good leadership is only one part of the solution albeit a crucial one to guide a diverse team to success and away from degenerating into ineffectiveness and/or rancor [55].

### 2.6. Failure to Establish Optimal Team Composition, Individual Roles and Dynamics, and Clear Roles for Members of the Team

Creating a high-functioning team is a challenging task. Most of the research on teams in medicine has been centered on patient care and/or specific patient conditions. Simulations have been developed to improve team performance in various clinical setting especially in emergency departments and in operating rooms [56,57]. The integration of different disciplines—physicians, nurses, and allied health professions within a single team has also garnered research attention. There may be inherent conflicts between physicians and nurse practitioners as they change their professional self-images in their responsibilities for patient care [58]. How a single physician or group of physicians function in a team within an organizational (non-clinical) structure has not been studied in depth. The management of a group of physicians in a health care organization requires different kinds of competencies from management of a clinical teams dealing with a patient [25]. Physician managers in health care teams may confront the “personal cost” of diminishing their professional roles as a physician [59]. This change in their self-perception may have implications on how the physician-manager functions in a physician or interdisciplinary team.

To be effective physician teams need to have (i) an understanding of and respect for the role of each person in the team; (ii) a recognition that it requires work to continue to have a functional team; (iii) a good understanding that the health care issues that the team is tasked with need managing; (iv) learned to work together; and (v) excellent communication between all team members [60]. A failure in any one of these five components will limit the ability to develop a high-performance team.

Teams may get “stuck”, become self-absorbed, or consumed with excess of negativity and destructive criticism [11]. A team can get locked into the same repetitive cycle, without meaningful new ideas [11]. Some teams that start as enthusiastic and successful teams degenerate in dispirited non-productive groups.

Constructing an “excessively” large team occurs as leadership may wish to ensure that all stakeholders are included and involved. While intuitively one may contend that if each team member has a specialized role, then the team will benefit from that expertise. There are, however, limits to the benefits of constructing a large team to be inclusive of multiple different kinds of specialized expertise. Team size influences the team productivity as the larger the size of the team, the greater is the probability that individual productivity will decline [61]. Large teams may limit the ability to be productive. The team needs to be of an optimal size to ensure relevant input and eventual “buy-in” to their recommendations.

### 2.7. Failure to Establish Psychological Safe Environment for Team Members

Psychological safety has been defined as the degree to which people view the environment as conducive to interpersonally risky behaviors like speaking out [8]. The level of psychological safety is more important than the level of education or years of experience of team members [62]. Teams with a climate of psychological safety that encourage high levels of participation, toward clear goals, become high-performance teams [25]. Each member of a team requires a safe environment to give an opinion without fearing of the consequences of expressing different opinions. It is incumbent on leadership to create a climate in which staff are inspired, supported, and motivated [27,63].

The lack of a psychologically safe environment is essentially an unfriendly workplace which in health services contribute to health care staff stress [64]. Dysfunctional workplaces impair the mental health of health care workers [64]. Dysfunctional workplaces may be accommodated by some physicians but for others it is a psychological distress. Focusing on the workplace as a social determinant of health suggests that for some physicians it is not burnout but rather a normal stress reaction to an uncongenial work environment [64]. It is important to recognize that organizational remedies for uncongenial stress are quite different from remedies to burnout [64].

There is a relatively little research on optimal methods to create a safe environment within the physician culture of an organization. Some physician groups have advocated for hospitals and health authorities to invest in developing the feedback skills (both giving and receiving) of administrative leaders as a way to promote psychological safety in health care teams [65]. The role of a high-performance organization is to educate medical leaders and potential leaders in this area [66]. Most importantly, the performance of these leaders needs to be evaluated with a crucial metric being the ability to create a workplace with minimal fear and maximal co-operation [66]. Providing medical leaders with feedback on their performance in this dimension along with the organization’s acceptance of the importance of this performance metric would contribute to creating a safer environment.

### 2.8. Failure to Address and Resolve Interpersonal Conflicts in Teams

An aspect of health care medical organization structure which can discourage development of a high-performance team for physician is interpersonal conflict between physicians, and interpersonal conflicts between physicians and allied health staff [67]. With respect to physicians, there are special issues. The assignment to medical leaders of the “power” of appointment and reappointment of medical staff, can not only impact a physician’s career in the institution but with other health care organizations that might be interested in hiring the physician. This “power” of the medical leader needs to be balanced within the organization or a culture of fear predominates [67,68]. A culture of fear not only destroys the capacity of the leader to lead a high-performance team but also destroys the desire of the team members to participate in the team.

“Even the best teams have tensions based on interpersonal differences, differences of opinion, competition, and other factors” [62]. Failure to address and resolve interpersonal conflicts can prevent the team from becoming a high-performance entity or worse leading to the disintegration of the team.

When an individual’s decisions are at variance with the majority of the team, the individuals that are in the minority may not respect the team’s decision [22]. If the individuals are physicians who are not accustomed to having their decisions questioned, the discord within the group may be greater.

### 2.9. Failure to Ensure Good Health and Well-Being of Physician Staff

Physician well-being or the lack of well-being, currently referred to as “physician burnout”, is now a well-recognized problem [69]. While the discrepancies in the definition of physician burnout limit accurate assessment of its prevalence, its existence and impact is generally accepted [70,71]. A “burned out” physician or one not performing optimally because of “burnout”, limits the maximum performance of the team. This concept has been supported by the finding that interventions for burnout reported significantly higher levels of teamwork in conjunction with attaining lower levels of burnout [72]. The interrelationship of burnout and team efficiency can be drawn from the field of family practice where studies have found that regardless of the team structure, family physicians who perceived their teams as being more efficient were less likely to be burned out [73]. Improving teamwork efficiency may be an effective strategy for enhancing team performance but also physician well-being [73].

In addition to the issue of burnout, a toxic workplace environment adversely affects physician health. Male physicians who reported abuse at work also reported significantly worse somatic symptoms along with less work satisfaction [74].

### 2.10. Failure to Ensure Physician Engagement with the Organization

Physician engagement can be viewed within the wider concept that an individual’s engagement in the organization, in which they work, improves organizational performance [75]. In addition, workers who say they are on a team are over twice as likely to be fully engaged than those who are not [76]. This finding is independent of demographics or work status [76]. Physician engagement is of considerable importance for the provision of high-quality patient care [77,78,79,80]. Higher levels of physician engagement are associated not only with improved patient care, lower patient mortality, but also with lower hospital costs [77,80,81].

There are a number of factors responsible for physician disengagement from their organization [82]. The engagement of physicians with an organization rests on physician’s expectation of the organization and their interest and desire to become engaged. It also involves the attitudes of the organization and its management “style”. A health care organization’s culture may not welcome physician opinions [81]. Another critical factor currently facing physicians is when the health care organization’s need for data in the form of charting and electronic medical records shifts more responsibility for the accuracy of medical record keeping onto the physician. As EMRs can be a source of frustration for physicians, physicians may become disenchanted with the institution that mandated the EMR [83]. Some organizations wish a level of “control” over physicians’ activities that create a master–servant relationship that leads to physician unhappiness. Thus factors associated with physician engagement involve individual characteristics, the work environment, and work outcomes [24,84]. However, physician engagement appears to be more a function of the organization and its leadership rather than the characteristics of the individual physician [85]. These and other issues of the organization may disengage physicians leading inevitably to a reduction in the “patient experience” [86] which is the antithesis of the organization’s values and vision statement.

Organizations that perform in the top quartile of having a “good” culture significantly outperformed those in the bottom quartile with respect to physician engagement, patient experience, and overall value-based purchasing performance [78]. Health care leaders have been extorted to pay attention to culture and to engage employees and physicians [78].

## 3. Overcoming Obstacles to Develop High-Performance Teams—Solutions

Before discussing the potential solutions to the obstacles to develop high-performance teams in health care, it is worth reiterating that heath care organizations are different from other types of organizations and these differences need to be taken into account in evaluation of any framework for performance [9]. Health care organizations have been viewed as complex adaptive systems [87]. Complex adaptive systems are “dynamic, massively entangled, emergent, and robust” [87]. This description fits health care organizations as they encompass a large number of participants with complex interrelationships that are entangled or enmeshed at different levels (Figure 1 shows only some of these). Health care organizations consist of a number of complex professional bureaucracies that deal with an even more complex set of political, legal, financial, customer (patient), and community challenges [9]. Because of the essential elements of health care, physicians and nurses have obligations and loyalty to a professional code of conduct, so that they cannot simply be ordered to comply with organizational dictums. These professionals must be convinced of the value of teamwork and the efforts to improve the culture of their health care organization.

**Figure 2:**
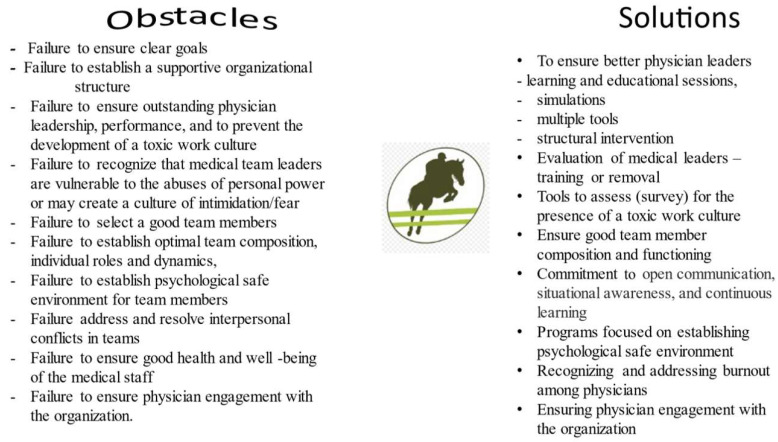
Graphical representation of the process of overcoming obstacles to develop high-performance teams involving physician in health care organizations.

Some of the obstacles, for developing high-performance teams, are relatively easy to fix such as ensuring clear goals for the organization and each of its teams (Figure 2). Some of the obstacles that involve personalities are more challenging. Training, education, and learning strategies for physicians as leaders and team members are of critical importance. The ge neral approach, in this area will be discussed first.

Most of the training for physicians in health care teams focuses on patient-centered activity and comparatively little is known on the role of physicians in a health care management team. However, a detailed review by Buljac-Samardzic et al. [88] provides information with regards to the kinds of training, tools, and programs that could be adapted or extended from the patient-centered model to a health care organization. Buljac-Samardzic et al. categorized approaches into three types—training, tools, and organizational design as well as a fourth approach that combines all the three elements [88].

In the training sphere, the crew resource management (CRM) approach has had mixed results or delivered a low to moderate quality level of evidence for benefit and has been usually aimed at nursing units in the organization [88]. The CRM programs rely heavily on patient simulators to train specific teamwork skills to physicians and other health professionals. The TeamSTEPPS (Team Strategies and Tools to Enhance Performance and Patient Safety) approach was designed to enhance teamwork skills and focuses on the delivery of quality and safe care. It promotes competencies, strategies, and the use of standardized tools on five domains of teamwork: team structure, leadership, communication, situational monitoring, and mutual support as well as focusing on change management, coaching, measurement, and implementation [88]. This approach leads to an improvement in some non-technical skills (teamwork, communication, safety culture) and some improvement in outcome—reduction in medical errors, as reduction in patient errors was the objective of this kind of training [88]. Simulation-based training utilizes techniques to approximate the actual patient experience with actors or mannequin patients and are centered on a clinical situation (e.g., cardiac arrest or shock) in order to improve technical and non-technical skills [89]. The Anesthesia Crisis Resource Management (ACRM) program is an example of a simulator-based programs [90]. These approaches have been tested mainly in acute patient care and demonstrate improvements mainly in technical skills [88].

In the field of tools or instruments that can be implemented independently to improve teamwork, the approaches include: structured communication technique such as SBAR (Situation, Background, Assessment, and Recommendation), (de)briefing checklists, and rounds [88]. Most but not all studies examining the SBAR approach found improvements in communication but only (very) low-level evidence studies were identified in the meta-analysis [88]. The objective of the briefing and debriefing approach is to create an opportunity for health care professionals to communicate and discuss issues often using a pre-specified list of questions or safety checklist. While this approach has shown some improvement in non-technical skills (e.g., communication, teamwork, safety climate) and objective outcome measures (e.g., reduced complications, errors, unexpected delays, morbidity), the studies are not consistent and some investigators are critical of its ability to produce long-term or sustained results and for its potential to create tension, and perpetuate a problematic culture [88].

Effective communication and teamwork is essential for the delivery of high quality, safe patient care [91]. Utilization of communication technologies not only permits exchange of patient information but also enhances the interaction of team members [88]; however, whether it is effective for building a high-performance team, independent of the patient safety sphere, is uncertain.

Studies of organizational (re)design have focused on payment systems, physical environment, standardization of processes in pathways and changing roles and responsibilities or forming dedicated teams for a certain unit or patient “disease” type [88]. Most studies found some improvements of non-technical skills; with this approach, however, a few found mixed results usually at a moderate to very low level of evidence [88].

A program approach that combines learning and educational sessions (e.g., simulation training, congress, colloquium), with multiple tools (e.g., rounds, SBAR), and/or structural intervention (e.g., meetings, standardization) maybe the most effective [88]. These programs have been developed mainly for patient safety such as the “comprehensive unit-based safety program” (CUSP) and include a tool kit for clinical teams [92]. The medical team training (MTT) program is based on a CRM approach but is inclusive of other types of approaches [93].

### 3.1. Overcoming the Failure to Ensure That the Goals, Purpose, Mission, and Vision Are Clearly Defined

From an organizational perspective, overcoming the general, non-specific statement about good or excellent patient care requires setting a health care institution’s goals (vision/mission) that provide more details on short-term performance metrics, intermediate project outcomes, and long-term aims that are realistic, match the intuition/community needs and time frame. This approach should enhance the organization’s performance.

At the level of individual teams within the organization, goals setting should take into consideration the objectives of more leadership that has formed or instructed the team. Negotiations may be necessary to eliminate ambiguity. The team must have a clear understanding of the goals and purpose of the team. It should be understood by all members of the team and especially its leader that there will be an evaluation of the team’s performance. The annual review of accomplishments should use clearly defined metrics known in advance by the entire team. The team should review its assessment and understand its accomplishments and failures to achieve its immediate goals and set the foundation for its intermediate and long-term goals.

### 3.2. Overcoming the Failure to Establish a Supportive Organizational Structure That Encourages High-Performance Teams

Changes in organizational structure are among the most challenging obstacles to overcome. Some changes can result from discussions within the hierarchical departmental structure. Competition for revenue, prestige, or ratings with a neighboring institution may lead to changes in the organization to create the environment that encourages high-performance teams.

Sometimes it is necessary to change the organizations culture to permit the advancement of high-performance teams. These kinds of changes can best be made by senior leadership. Lessons can be learned from the importance of people management in improving clinical practice within hospitals [94]. The importance of the three elements, development of teamwork, performance management, and sophisticated training cannot be underestimated [94]. Good people management can change the work culture [94], which in turn will create a high-performance medical organization.

### 3.3. Overcoming the Failure to Ensure Outstanding Physician Leadership

One method to overcome the failure to ensure outstanding physician leadership, which is critical for a high-performance team, is to focus attention directly at developing programs for medical leaders [95] to create high-performance teams. An approach that combines learning and educational sessions, simulation with multiple tools, and/or structural intervention can ensure better physician leaders. These programs need to ensure that medical leaders become skilled in learning how to participate in the development of clearly defined team objectives, encourage high levels of participation from team members, as well as ensuring a commitment to excellence and support for innovation [63].

The leader must also manage conflict, ensuring a productive balance between harmony and healthy debate [14,27]. Programs need to ensure that medical leaders are evaluated on their ability to encourage, inspire, and lead a high-performance team. In virtually all other professional venues where supervision is important for outcomes (e.g., business, sports), leaders are evaluated regularly on the efficacy of their supervision skills. The process of evaluation of medical leaders has been implemented in several leading medical organizations such as the Mayo Clinic [66]. More general “360” reviews of physician leaders are an important step forward [96] but do not necessarily focus on evaluation of their leadership ability to develop and led a high-performance team.

### 3.4. Overcoming the Failure to Recognize That Team Leaders Are Vulnerable to the Abuses of Personal Power or May Create a Culture of Intimidation/Fear, and a Toxic Work Culture

It is extremely important to reiterate that health care environment can readily become a culture of intimidation and that it is a global and not a regional phenomenon restricted to only certain countries [36,37,38,39,40,41]. Bullying in the medical workplace, is not restricted to only one specialty or level of training. The victim can be a trainee, a physician in practice, or a consultant [43,44]. The bully is most often a superior [43,44], who does not have the capacity to create a safe environment which encourages creative ideas and their expression. A team with this kind of leader will not coalesce into a high-performance team.

Programs that combine learning and educational sessions with multiple tools and/or structural intervention should be compulsory for individuals before they take on leadership positions and for their reappointment. Annual surveys of the physician staff, conducted by outside personnel, need to incorporate questions specifically whether the physician believes that the person in leadership has acted in a manner that is consistent with being a bully or has created a culture of intimidation/fear. Such reports should go to all levels of leadership including the person being reviewed. Bullies should be retrained or removed and should not be the mentors of the next generation of medical leaders.

Tools to assess (survey) for the presence of a toxic work culture should be routinely be used in the organization [47] by an outside body that reports to the board. When it becomes apparent that a toxic work culture exists in section of the organization or the entire organization, action should be taken immediately to correct this problem.

### 3.5. Overcoming the Failure to Select a Good Team and Team Members—Team Members Who Like to Work in Teams or Are Willing and Able to Learn How to Work in a Team and Ensure a Well-Balanced Team Composition

Inviting physicians to participate in a team must include providing potential team members with the duties and responsibilities of membership. Brief written statement should be submitted by the individuals outlining their background, interests, and accomplishments that make the ideal for team membership. Too often a physician is just “tapped on the back” and asked to join a team. Interviewing prospective candidates for committee membership and obtaining input of others who have worked with the individual contribute greatly to the selection process for a team member just as it does for selection of a person for a job in the organization.

### 3.6. Overcoming the Failure to Establish Optimal Team Composition, Individual Roles and Dynamics, and Clear Roles for Members of the Team

A meta-analysis evaluating a large number of teams concluded that training of the team is invaluable for organizations to enhance outcome especially on the cognitive, outcomes, teamwork processes, and performance outcomes [97]. Considered all outcomes in that meta-analysis, team training was most effective to improve team processes [97]. It bears repeating that physicians have usually not undergone this kind of training in medical school or residency. Processes such as communications and coordination are improved by training teams and this training will lead to improvement in decision-making that should ultimately improve team performance [97].

Building effective teams requires not only the delineation of clear goals, but also an understanding of each member’s role in reaching that goal, and continuous feedback as issues are identified. The solo mentality required to become a health care provider can be modified. Consistent buy-in and support from senior administration to deal with disruptive personalities when present in the team is vital for long-term success [98].

In addition to a general educational approach to medical leadership, it is valuable to have a program approach that combines learning and educational sessions including simulation training, educational sessions, with multiple tools including SBAR, and/or structural intervention [88] should be effective in overcoming failure to establish optimal team composition and dynamics, and clear roles for members of the team. For example, TeamSTEPPS tools [56,57], were associated with statistically significant improvements in teamwork metrics in an academic interventional ultrasound practice with the most notable improvements occurring in communication among team members and role clarification [99].

Several approaches based on techniques used in other fields may be helpful. One proposal is to include learning the psychological construct of transactional analysis to help physicians develop insight into and optimize the use of different communication styles [4]. Another technique from a basic science approach is concept mapping. Concept mapping is a useful graphical technique to identify knowledge gaps [100]. It may help some disciplines required to create and develop high-performance teams [100].

Clinical teams have realized the benefit of commitment to the concepts of open communication, situational awareness, and continuous learning [101]. The ability of teams to communicate well needs to be assessed and where deficient they need to be improved [102]. The greater the interdependence and the closer the co-operation, the higher the efficiency and the better the work climate for the team [103].

Good leadership is required to identify and achieve agreed upon outcomes and to enhance the overall performance of the team and physician team members must have a commitment to working collaboratively [60]. The basic requirement is to ensure a common vision and universally agreed upon goals for the team. An unattainable goal is both frustrating and disengaging for a team member and will eventually lead to disintegration of their motivation to be on the team.

### 3.7. Overcoming the Failure to Establish Psychological Safe Environment for Team Members

Programs that are focused on the necessity to establish psychological safe environment for team members and the best approaches to establish a safe environment can be learned through a combination of educational sessions, simulations, and structural intervention. These kinds of programs should be compulsory for individuals before they take on leadership positions and for reappointment in such positions. It is worth reiterating that organizational remedies to uncongenial stress are quite different from remedies to burnout [64].

There is a relatively little research on optimal methods to create a safe environment within the physician culture of an organization. Some physician groups have advocated for hospitals and health authorities to invest in developing the feedback skills (both giving and receiving) of administrative leaders as a way to promote psychological safety in health care teams [65]. The role of a high-performance organization is to educate medical leaders and potential leaders in this area [66]. Most importantly, the performance of these leaders needs to be evaluated with a crucial metric being the ability to create a workplace with minimal fear and maximal co-operation [66]. Providing medical leaders with feedback on their performance in this dimension along with the organization’s acceptance of the importance of this performance metric would contribute to creating a safer environment.

The implementation of TeamSTEPPS tools was associated with statistically significant improvements in safety and teamwork metrics in an academic interventional ultrasound practice [99]. The most notable improvements were seen in communication among team members and role clarification. This model, which has been successfully implemented in many non-radiologic areas in medical care, was also applicable in imaging practice [99].

### 3.8. Overcoming the Failure to Address and Resolve Interpersonal Conflicts in Teams

Recognizing that interpersonal conflicts occur in teams is a first step in preventing and overcoming the destructive effects of these conflicts. Training for each member of the team should be mandatory.

An essential component to overcome and resolve interpersonal conflicts in teams is to have medical leaders and all team members appreciate the destructive nature of interpersonal conflicts. Medical leaders should be evaluated on their ability to diffuse and repair interpersonal conflicts.

### 3.9. Overcoming the Failure to Ensure Good Health and Well-Being of Physician Staff

As we noted previously physician burnout is a major problem for the health care system [69,70,71]. A “burned out” physician or one not performing optimally because of “burnout” limits the maximum performance of the team which he/she is part of. Physician burnout needs to be addressed at the level of the health care organization, individual teams, and the individual level [72].

Organizational strategies as well as individual-focused programs can produce meaningful reductions in burnout among physicians [104]. These strategies must be implemented within the organization and cannot consist only of adding “physician engagement” as another portfolio to an overworked hospital or medical leader. Neither should it devolve to only setting up a group social function but rather must involve a thorough change in the organization management and culture. Organizational strategies to permit workflow redesign, improve communication, especially among clinicians and staff, and develop quality improvement projects to address clinician’s concerns can reduce physician burnout and work place dissatisfaction [105]. In some cases amelioration of a specific organizational stressor, for example the interaction with the electronic medical record, may alleviate some elements of physician burnout [106,107]. Organization-directed workplace interventions that improve electronic processes, programs aimed at restoration of positive emotions around work in health care institutions can reduce burn-out [108].

### 3.10. Overcoming the Failure to Ensure Physician Engagement with the Organization

There are several pathways to ensure physician engagement with the organization. First, is the correction of organizational characteristics that contribute to disengagement as well as stress and burnout [109] should increase physician engagement in efforts to develop a high-performing team.

Second, is the recognition there must be relevant and optimal avenues for the organization and its operations teams and administration hierarchies to interact with the physicians or medical staff of the institution. In order to accomplish the mission/goal of meaningful physician engagement, one approach that we utilized was to establish a “charter” for physician engagement [82]. Based in part on the conceptual schema from The International Association of Public Participation’s IAP2 Spectrum [110], we defined our concept of meaningful physician engagement and customized the engagement spectrum construct for physician of our medical staff association with our health care authority, hospital, and community. The engagement spectrum spans the range from recognizing that there is a spectrum starting with the basic level of “*informing*” that does not require physician involvement, to *consulting* physicians on draft plans, to *involvement* of physicians in the planning process, to *collaboration* when physicians share decision-making with the institution, and to *empowering* where physicians identify issues create the solutions and act with institutional support to accomplish the solution [82].

Third, institutions that wish to increase the engagement of physicians in improving clinical and financial performances through medical leadership should focus on selecting and developing leaders who are strong strategists (who prioritizes the interests of the hospital by participating in hospital strategy and decision making), socially skilled (strong collaboration and communication skills), and who are accepted by clinical peers [111]. While credibility among medical peers appeared to be the most important factor for medical leadership, of paramount importance is the ability to contribute to strategic thinking and institutional decision-making to ensure credibility among senior leadership in the organization [32].

## 4. Conclusions

**Figure 3:**
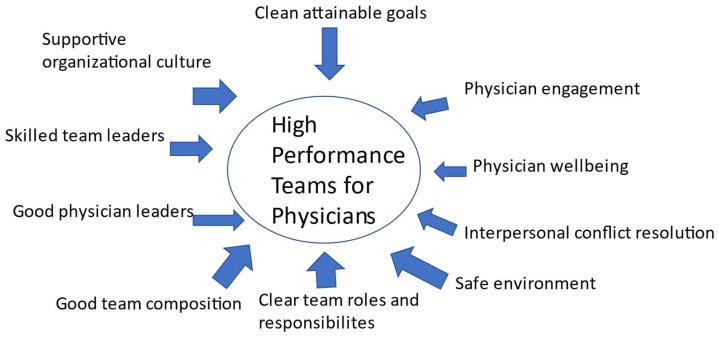
The critical dimensions for creating a high-performance team with physicians, in health care organizations.

There are many challenges for health care organizations to develop, or improve their work place but the creation of high-performance teams is one of them. Identifying and mitigating the factors inhibiting physician participation and leadership in a health care team should lead to better performance in patient care. The major limitation of this study is that it was dependent on available literature which did not necessarily have experimental evidence on each of the components of the obstacles as it applied to physicians. We have also outlined a number of areas that require additional research and outcome trials. Some health care organizations have been considered to “stand at the nexus of an unstable political and socioeconomic landscape” [4]. The construct developed herein (Figure 3) advances the concept of ten critical dimensions for creating high-performance teams with physicians, in health care organizations. The success of health care organizations may well reside on their ability to develop frameworks that encourage physician engagement, innovation while nurturing a safe environment with open communication, devoid of bulling and harassment, with clear goals with a supportive organizational structure that encourages high-performance teams. All health care organizations should benefit from overcoming obstacles to develop high-performance teams.

## Data Availability

Not applicable.
